# Mesoscale Metabolic Channeling Revealed by Multimodal Microscopy

**DOI:** 10.21203/rs.3.rs-4096781/v1

**Published:** 2024-04-17

**Authors:** Rafael Arrojo e Drigo, Aliyah Habashy, Christopher Acree, Keun-Young Kim, Thomas Deerinck, Emilee Patterson, Louise Lantier, Owen McGuinness, Mark Ellisman

**Affiliations:** Vanderbilt University; Vanderbilt University; Vanderbilt University; University of California San Diego; University of California San Diego; Vanderbilt University; Vanderbilt University; UCLA

**Keywords:** glucose metabolism, organelle contacts, tissue organization, cell architecture

## Abstract

Metabolic homeostasis within cells and tissues requires engagement of catabolic and anabolic pathways consuming nutrients needed to generate energy to drive these and other subcellular processes. However, the current understanding of cell homeostasis and metabolism, including how cells utilize nutrients, comes largely from tissue and cell models analyzed after fractionation. These bulk strategies do not reveal the spatial characteristics of cell metabolism at the single cell level, and how these aspects relate to the location of cells and organelles within the complexity of the tissue they reside within. Here we pioneer the use of high-resolution electron and stable isotope microscopy (MIMS-EM) to quantitatively map the fate of nutrient-derived ^13^C atoms at subcellular scale. When combined with machine-learning image segmentation, our approach allows us to establish the cellular and organellar spatial pattern of glucose ^13^C flux in hepatocytes *in situ*. We applied network analysis algorithms to chart the landscape of organelle-organelle contact networks and identified subpopulations of mitochondria and lipid droplets that have distinct organelle interactions and ^13^C enrichment levels. In addition, we revealed a new relationship between the initiation of glycogenesis and proximity of lipid droplets. Our results establish MIMS-EM as a new tool for tracking and quantifying nutrient metabolism at the subcellular scale, and to identify the spatial channeling of nutrient-derived atoms in the context of organelle-organelle interactions *in situ*.

## Introduction

Tissue function is supported by cell metabolism pathways that are modulated to meet changes in nutrient availability and energetic demands that occur throughout an organism’s lifetime. Much of our knowledge regarding cell metabolism is derived from bulk metabolomics using stable or radioactive isotopes (i.e., ^13^C and ^14^C, respectively). Throughout the years, this approach has revealed differences in how cells utilize nutrients to maintain energy and cell homeostasis during different cell states ^1,2^ and how these aspects are impacted by aging, cancer, degenerative, and/or metabolic diseases ^3^. Cells are organized in sub-cellular compartments created by organelles that handle essential processes necessary for cell function, such as mitochondrial respiration or protein synthesis within the endoplasmic reticulum (ER) and Golgi apparatus ^4,5^. Several aspects of cell metabolism require proper organization of organelle-organelle-interaction networks that create distinct intracellular compartments such as mitochondria-ER or mitochondria-lipid droplets contact sites ^6–7^. These sub-cellular compartments are dynamic and interact via proteins that mediate membrane anchoring and/or the exchange of molecules and ions between organelles ^6–9^. Perturbation of these organelle contact sites disrupts cell and whole-body metabolism and have been linked to the patho-physiology of metabolic and neurodegenerative diseases ^8,10–13^. Therefore, there is a need to study and understand the principles that guide the spatial organization pattern of cells and organelles *in situ* and their correlation to changes in animal and cell metabolism.

Different super resolution light and electron microscopy techniques have been applied to determine the architecture and spatiotemporal dynamics of organelle-interaction networks with nanometer resolution ^7^. Moreover, recent advances in imaging metabolomics techniques such as MALDI-MS ^14^ and ToF-SIMS ^15^ allowed the visualization of the spatial distribution pattern of metabolites and molecular flux at tissue and multi-cellular scales. However, these techniques are unable to detect and measure metabolites at sub-cellular resolution so that one can study the correlation between cell metabolism and cell and/or organelle anatomies. In recent years, we have developed a new correlative microscopy pipeline that combines high-resolution scanning electron microscopy (SEM) with multi-isotope mass spectroscopy (MIMS) that is called MIMS-EM ^16^. MIMS-EM leverages SEM’s high spatial resolution and the high-resolution mass detectors of MIMS to simultaneously detect and quantify stable isotope incorporation (e.g., ^15^N, ^14^N, ^13^C, or ^12^C) into macromolecules to create spatially annotated maps of stable isotope flux overlaid with (intra)cellular architecture. We have previously applied MIMS-EM and stable isotope-labelling of animal tissues and cells to create quantify the age of multiple biological structures, from protein super-complexes to organelles to cells ^16–18^. These results revealed the vast heterogeneity of age and longevity of biological structures in a multi-scale phenomenon we refer to as age mosaicism ^16^.

Here, we apply MIMS-EM to annotate the spatial channeling of atoms derived from nutrient metabolism at animal, tissue, cell, and intracellular scales. This is achieved by combining MIMS-EM with stable isotope labelling of mice (SILAM) using [U-^13^C_6_]-glucose tracers, *in vivo* animal metabolism measurements, and gas-chromatography mass spectrometry (GC-MS) to extract multiple indexes of glucose metabolism and flux across scales. Using deep-learning (DL)-based image segmentation and spatial analysis tools, we map the subcellular location of individual organelles and chart the landscape of organelle-interaction networks to quantify subcellular changes that occur in response to increases in circulating glucose levels. This uncovered the association of enzymes involved in glycogen synthesis with lipid droplets, and the existence of two subpopulations of ER-interacting mitochondria marked by distinct glucose-derived ^13^C enrichment and ER interaction patterns. Together, our approach establishes a multi-modal framework to study the spatial landscape of nutrient flux and organelle organization to reveal sub-cellular organization patterns of enzymes and organelles involved in glucose metabolism.

## Results and Discussion

### In vivo labelling of mice using [U-^13^C_6_]-glucose and whole-body metabolomics.

**Figure 1:**
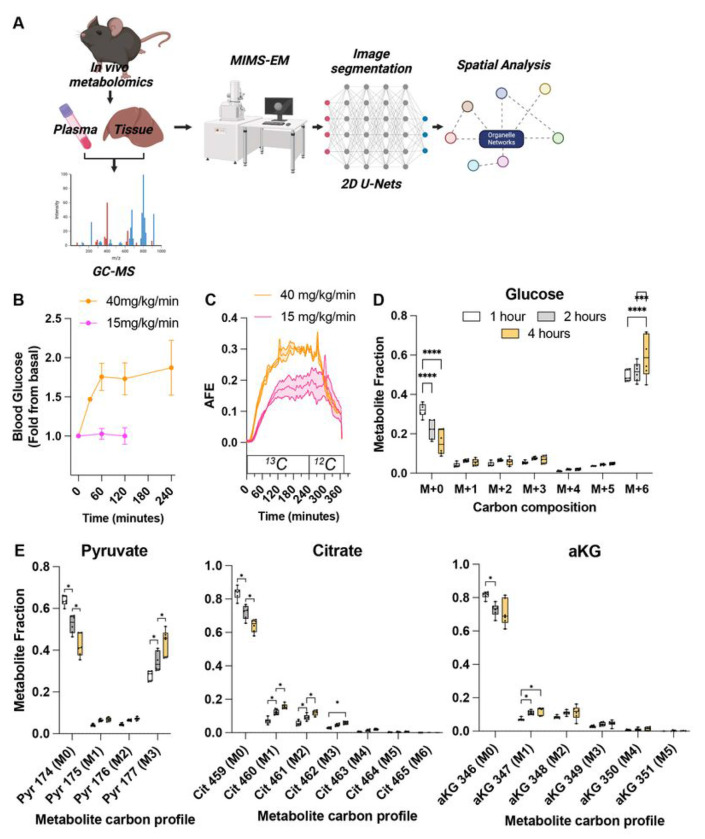
*In vivo* labelling of mice using [U-^13^C]-glucose. **(A)** Illustration of the approach used to label freely moving and unanesthetized mice with [U-^13^C]-glucose for up to 4 hours using catheters followed by MIMS-EM and spatial analysis. **(B)** Blood glucose levels measured from mice continuously infused with 15 or 40mg/min/kg of total body mass for up to 4 hours. **(C)** Expelled ^13^CO_2_ (in parts per million (ppm)) measured from the atmosphere of custom-made metabolic cages using gas mass spectrometers. ^13^C glucose was infused for the first 240 minutes and replaced with ^12^C glucose for an additional 120 minutes. **(D-E)** GC-MS analysis to determine the fractional ^13^C enrichment of circulating glucose molecules and of secondary metabolites Pyruvate, Citrate, and alpha ketoglutarate (aKG) generated from glucose metabolism. Each dot represents an animal. In (B), data shown as ± standard deviation.

To measure the flux of glucose from whole body to the organelle level and its correlation with cellular and organelle organization, we created a multi-modal pipeline combining the delivery of [U-^13^C_6_]-glucose with tissue stable isotope mass spectrometry and MIMS-EM (Figure 1A). We delivered [U-^13^C_6_]-glucose to freely moving and awake animals using intra-venous catheter that contained an additional port for arterial blood sampling to quantify blood glucose and plasma metabolite levels and ^13^C enrichment in those metabolites (Figure 1A). First, we placed 6-hour fasted 8-week-old male C57/BL6J mice inside metabolic cages and continuously infused each animal with 15 or 40 mg·kg^−1^·min^−1^ of [U-^13^C_6_]-glucose for up to 4 hours. These doses were chosen to evaluate *in vivo* glucose metabolism rates in response to glucose dosages that either matched or exceeded the rate of endogenous glucose production in mice ^19^. As expected, mice infused with 15 mg·kg^−1^·min^−1^ remained normoglycemic, while mice dosed with 40 mg·kg^−1^·min^−1^ experienced a sustained increase in glucose concentration (Figure 1B). Next, to investigate the kinetics of whole body [U-^13^C_6_]-glucose oxidation *in vivo*, we measured the relative enrichment of ^13^C in the expelled breath CO_2_ using stable isotope mass spectrometer gas detectors coupled to our metabolic cages (Figure 1C, and Figure S1A). This approach quantified time- and dose-dependent increases in ^13^CO_2_ in [U-^13^C_6_]-glucose-infused mice, thus confirming that [U-^13^C_6_]-glucose molecules were delivered and oxidized within the first 60 minutes and reached a plateau within 120min (Figure 1C). Accordingly, exchange of [U-^13^C_6_]-glucose for unenriched glucose caused ^13^CO_2_ to quickly fall over time (Figure 1C). Similar results were observed in mice exposed to a longer 16-hour fast and infused with 40 mg·kg^−1^·min^−1^ of [U-^13^C_6_]-glucose, including increased insulin release (Figure S1B-D), thus validating our stable isotope delivery and quantification of [U-^13^C_6_]-glucose oxidation rates *in vivo*.

In response to an increase in blood glucose, pancreatic beta cells secrete insulin to normalize blood glucose levels ^20^. Insulin acts on skeletal muscle depots that metabolize glucose into secondary metabolites that can be measured in the circulation (i.e., lactate and pyruvate), and stimulates the liver and adipose tissue to store glucose-derived carbons into large macromolecules such as glycogen or triglycerides, respectively. To investigate the amount of [U-^13^C_6_]-glucose and ^13^C-labelled glucose derived metabolites, we performed GC-MS on plasma samples collected during our infusion experiments. This identified a gradual and significant decrease in the fractional abundance of ^12^C_6_-glucose (M+0) and an increase in ^13^C-labelled glucose (M+6) (Figure 1D), with a similar pattern in the appearance of M+3 being observed for several circulating metabolites such as lactate, pyruvate, glycerol, and alanine (Figure 1E, Figure S1D-G, and Supplementary Table 1). These results indicate that as [U-^13^C_6_]-glucose floods the circulatory system ^13^C abundance in glucose (M+6) and glucose-derived secondary metabolites (M+3) increase.

### Quantification of glucose-derived ^13^C incorporation at tissue and cell scales.

The liver is a key organ in the glucose homeostasis response, where hepatocytes are organized in distinct architectural zones with unique transcriptional and metabolic profiles that underlie differences in glucose metabolism and glycogenesis ^21,22^. Therefore, we used the liver as a benchmark to investigate the flux of glucose-derived ^13^C atoms at the tissue scale. To validate our approach and to confirm ^13^C enrichment in newly synthesized glycogen molecules, we performed GC-MS of isolated glycogen molecules from mice infused with 40 mg·kg^−1^·min^−1^ [U-^13^C_6_]-glucose after an overnight fast. We observed a gradual increase in ^13^C-labelled glycogen molecules, as expected (Figure S2A).

**Figure 2:**
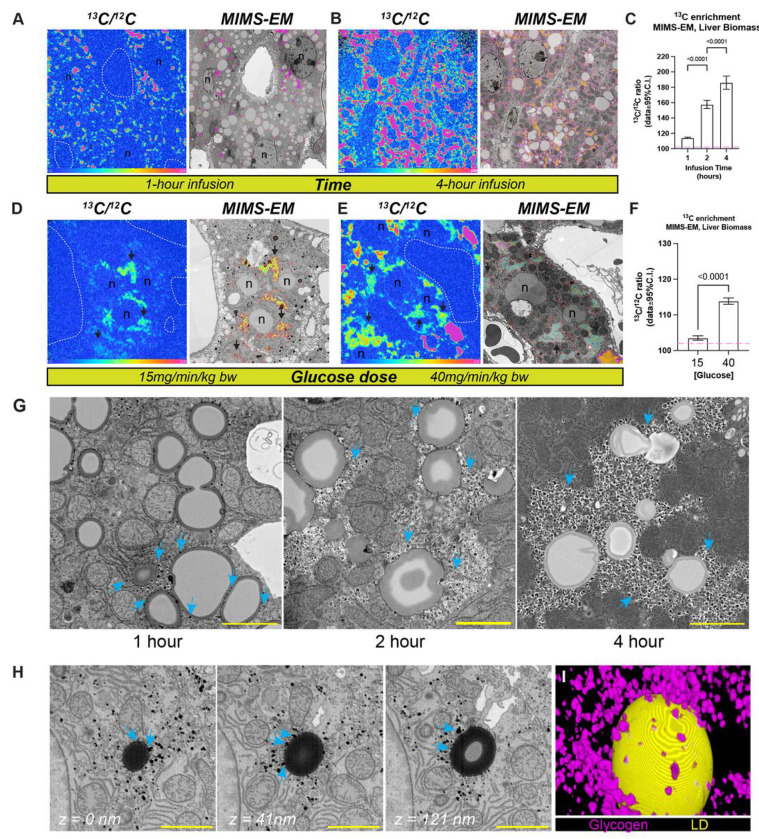
Quantification of liver tissue ^13^C levels using MIMS-EM. **(A-B)** Correlated ^13^C-to-^12^C (^13^C/^12^C) ratiometric images acquired using MIMS and registered to scanning electron microscopy (SEM) of hepatocytes to create MIMS-EM maps. Data from mice continuously infused with [U-^13^C]-glucose at 15 or 40mg/min/kg of total body mass for 2 hours. **(C)** Quantification of ^13^C/^12^C ratios in the biomass of mice shown in (A-B). **(D-E)** MIMS ^13^C/^12^C ratiometric images registered to hepatocyte SEM micrographs to create MIMS-EM maps. Data from mice continuously infused with 40mg/min/kg of [U-^13^C]-glucose for 1, 2, or 4 hours. **(F)** Quantification of ^13^C/^12^C ratios in the biomass of mice from shown in (D-E). **(G)** Representative SEM micrographs showing clustering of glycogen crystals around lipid droplets (LDs) in mice at 1- and 4-hour timepoints. Blue arrows point to glycogen depots. **(H)** Serial slices extracted from a previously published volumetric EM dataset centered in a lipid droplet. Relative distance in the z-axis from the first section are shown in white. Blue arrows indicate glycogen depots in the periphery of the lipid droplet. **(I)** 3D reconstruction rendering of the volume shown in (G). In (A-B and D-E), magenta and orange colors represent lower and higher different levels of ^13^C enrichment, respectively. In (G-H), scale bar = 2 microns. In (C) and (F), *p*-values are displayed in-graph.

We applied MIMS-EM to quantify the flux of glucose-derived ^13^C atoms in hepatocytes from mice infused with [U-^13^C_6_]-glucose and focused on hepatocytes close to the central vein because of their higher potential to channel glucose towards glycogenesis ^22^. MIMS-EM imaging collected data for multiple isotopes (i.e., ^13^C, ^12^C, ^32^S, and ^14^N) that were co-registered on high-resolution hepatocyte micrographs previously acquired using SEM ^16^. Briefly, processing of MIMS-EM data requires the alignment of MIMS and SEM images in a process that involves mapping of fiducial points for cross-platform image registration (Figure S2B) ^17^. These steps are required for the correlative nature of MIMS-EM since MIMS imaging causes significant deformations in the X and Y axes (Figure S2C-D) that must be corrected for to achieve a high degree of true image overlap (Figure S2E-F) ^17^. MIMS-EM of hepatocytes revealed time- and dose-dependent accumulations of ^13^C within the total hepatocyte biomass (Figure 2A–F, Figure S3A), which is consistent with incorporation of glucose-derived ^13^C into cellular structures and macromolecules. Spatial enrichment and distribution of hepatocyte ^13^C was characterized by a granular cytosolic architecture that largely co-localized with glycogen stores, thus indicating that these depots contained newly synthesized glycogen molecules identified with bulk GC-MS (Figure 2A–F, Figure S2A). Quantitative analysis of hepatocyte SEM micrographs revealed a time-dependent growth of glycogen depots correlated with glycogen ^13^C enrichment quantified with MIMS-EM (Figure S3A-C). Next, to place these results in a tissue- and cell-type-specific context, we applied MIMS-EM to monitor ^13^C flux in brown adipocytes. Brown adipocytes contain small multilocular lipid droplets that interact with a dense mitochondrial population engaged in oxidative and glycolytic glucose metabolism pathways that generate energy and replenish LD content ^23^. MIMS-EM of brown adipocytes revealed ^13^C enrichment in LDs and little-to-no enrichment in cytosolic, mitochondrial, or nuclear regions, thus indicating that these cells channeled glucose-derived ^13^C into saturated fatty acid synthesis (Figure S3D). ^13^C-labelled glycogen stores observed within 1 hour of glucose infusion were in direct contact and/or within the immediate neighborhood of lipid droplets (LDs), which became engulfed by glycogen over time (Figure 2A and 2G). Similar results are observed in three-dimensional (3D) reconstructions of previously published volumetric electron microscopy of adult mouse hepatocytes^12^ (Figure 2H–I), thus suggesting that enzymes involved in the glycogenesis process could be tethered to the scaffold of LDs.

Together, this data demonstrates how in vivo metabolic tracing and MIMS-EM can be combined to quantify glucose carbon flux at cell scales to identify cell type and intracellular sites involved in nutrient channeling towards glycogenesis or LD synthesis in a tissue-specific manner.

### The subcellular architecture of ^13^C flux in hepatocytes.

**Figure 3:**
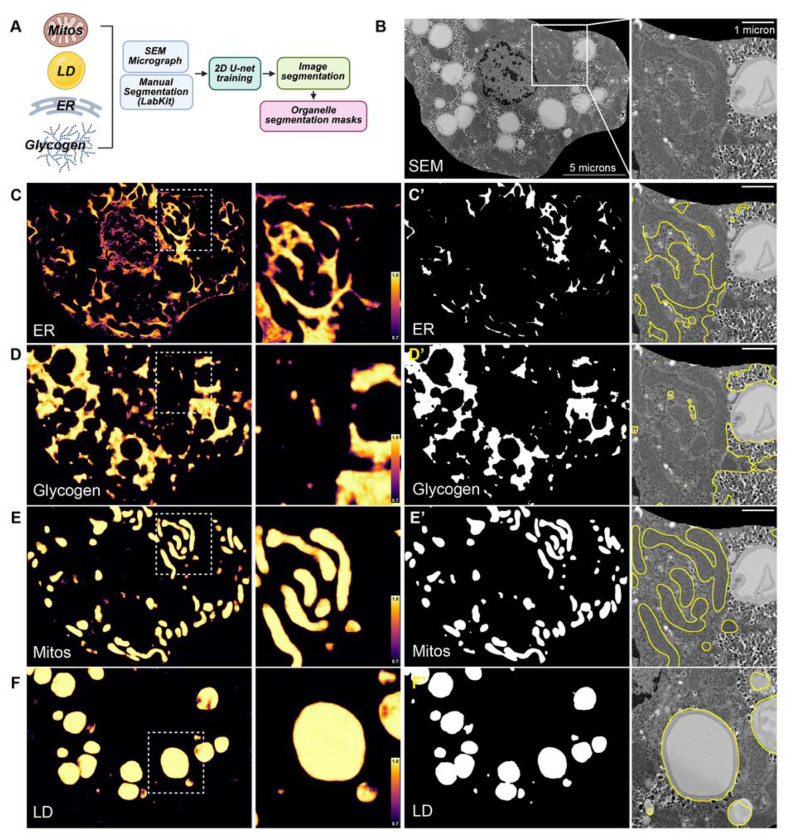
Automatic segmentation of organelles in MIMS-EM datasets. **(A)** Illustration of our organelle segmentation pipeline. A sub-set of SEM images are manually segmented to and used to train a 2D U-net model. This organelle-specific model is applied to SEM micrographs to create organelle segmentation masks that are used for downstream data analysis. **(B)** SEM micrograph of a single hepatocyte. Right, zoomed-in region of the cell marked by the white dashed square in (B) to highlight clustered mitochondria, lipid droplet, and glycogen. Cell from a mouse infused with 40mg/min/kg of [U-^13^C]-glucose for 4 hours. **(C-F and C’-F’)** Pixel classification confidence maps created by 2D U-nets trained to segment endoplasmic reticulum (ER), glycogen, mitochondria (Mitos), and lipid droplets (LDs), respectively. Here, each pixel receives a score of 0 to 1, where 1 indicates 100% confidence that pixel is correctly classified by a given DL model. In (C-F), raw data from DL-segmentation of the cell shown in (B), including zoomed-in region. Color scale bar represents the segmentation confidence interval from 70-to-100% model confidence. In (C’-F’), binarized organelle segmentation masks after 2D U-net segmentation and image processing and overlay on SEM data. Overlays are shown for the zoomed in quadrant highlighted in (B).

Changes in organelle architecture and organelle interaction networks can affect several aspects of cell function and whole-body metabolism ^6,10,13,24^. Besides identifying the intracellular location of glycogen synthesis, MIMS-EM of hepatocytes revealed that ^13^C accumulation can also occur in cytosolic spaces devoid of glycogen that contained mitochondria, ER, and/or LDs (Figure 2A and D). This suggested that glucose-derived ^13^C could be channeled towards and/or accumulate in other regions of the cell, which in turn could have distinct organelle distribution landscapes and interactomes. To test this hypothesis, we created a computational framework to map the spatial organization of individual organelles and their physical contacts with neighboring organelles to reconstruct organelle-specific interactomes correlated with movement of ^13^C within hepatocytes at the single cell level. To achieve this, we trained 2D U-nets to segment hepatocyte mitochondria, LDs, ER, and glycogen compartments (Figure 3A–B). Our organelle segmentation tools were benchmarked against a representative subset of manually annotated SEM images to create organelle classifiers with at least 90% confidence and a < 5% false positive organelle identification rate (Figure 3B–F, Figure S4A-B).

**Figure 4:**
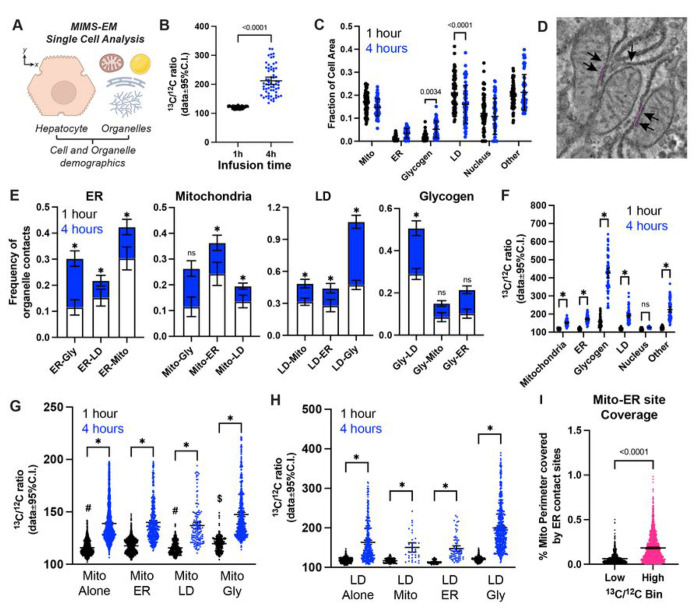
Spatial annotation of hepatocyte anatomy and ^13^C enrichment patterns. **(A)** Cartoon illustration showing the single cell analysis of MIMS-EM data. **(B)** Hepatocyte ^13^C/^12^C ratios from mice continuously infused with 40mg/min/kg of [U-^13^C]-glucose for 1 or 4 hours. **(C)** Relative fraction of hepatocyte cell area occupied by endoplasmic reticulum (ER), lipid droplets (LDs), glycogen, mitochondria, nucleus, or other cytosolic elements. Data from n=3 mice per group, from mice continuously infused with 40mg/min/kg of [U-^13^C]-glucose for 1 or 4 hours. **(D)** Close up of mitochondria and ER organelles. Magenta line annotates the location of Mitochondria-ER contact sites. Black arrows point to mitochondria-ER contact sites. **(E)** Relative frequency of organelle-contact types after 1 or 4 hours of [U-^13^C]-glucose infusion. **(F)** ^13^C/^12^C levels by type of organelle after 1 or 4 hours of [U-^13^C]-glucose infusion. **(G-H)** ^13^C/^12^C levels by different types of mitochondria or LDs, respectively, classified by the identity of their closest interacting partner after 1 or 4 hours of [U-^13^C]-glucose infusion. **(I)** Percentage of mitochondrial perimeter covered by ER contact sites in mitochondria with lower or higher ^13^C/^12^C ratios as shown in Figure S5D. In (B, C, and F), each dot represents data from a single cell, and * p < 0.01. Data shown as ± 95% of the confidence interval of the mean.

Using this approach, we extracted the X and Y coordinates of individual organelles and quantified their morphological and ^13^C-enrichment levels in hepatocytes (Figure 4A–C). This revealed significant changes to the overall hepatocyte organelle composition and ^13^C-enrichment in response to an acute and sustained increase in circulating glucose levels, largely limited to a decrease in LD-occupied area and a large increase in glycogen stores (Figure 4C). This increase in glycogen reflects the active storage of glucose-derived C into glycogen chains, and loss in LDs is explained by suppression of lipolysis and subsequent decrease in fatty acid delivery to the liver that combined by a relative maintenance in the secretion of triglycerides in VLDL particles ^25^. Changes in whole animal and/or cell metabolic demands are associated with reorganization of organelle-organelle contact sites and organelle interaction networks ^12,13^. To investigate how these aspects are regulated as hepatocytes synthesize glycogen and store glucose-derived ^13^C, we created a contact-search computational framework that marks the position of organelle contact sites in MIMS-EM datasets. This is achieved by a vector-based search for neighboring pixels by overlapping organelle segmentation masks to identify likely areas of organelle contact located within 5-to-10 nanometers in distance (Figure 4D and Figure S5A). This allowed us to estimate changes that occur to organelle contact site size and frequency for mitochondria, ER, LDs, and glycogen depots at the single cell level (Figure 4E, Figure S5B). This approach revealed that ~25-30% of all mitochondria, ER, and LDs are in contact with each other within the first hour (Figure 4E). Within 4 hours, these connections are lost to increases in organelle interactions connections with the growing glycogen mass, particularly for ER and LDs (Figure 4E). This was also characterized by a significant decrease in the area occupied by organelle-contact sites, thus indicating that these organelles moved away from each other as glycogen is synthesized near LDs (Figure S5B).

Next, to investigate how these changes in organelle-contacts correlate with overall organelle ^13^C enrichment, we quantified the ^13^C/^12^C ratios in mitochondria, ER, LD, and glycogen and found that all these compartments become significantly enriched with ^13^C over time (Figure 4F and Figure S5C). Here, glycogen depots have the highest levels of enrichment (as expected given the much higher fractional turnover rate of the glycogen pool), followed by ER, LD, mitochondrial, and other cytosolic compartments (Figure 4F). Moreover, histogram analysis of organelle ^13^C/^12^C ratios revealed that ER and glycogen had relatively homogeneous enrichment ^13^C/^12^C ratios, whereas mitochondria and LDs had a clear bi-modal distribution pattern within the first hour of glucose infusion (Figure S5C), thus suggesting the existence of organelle sub-populations. Organelles function inside the cell can be heterogeneous and dependent on the nature of their organelle-organelle contacts ^6^. For example, in hepatocytes, LD-associated mitochondria have distinct protein expression patterns and are more adept for fatty acid oxidation versus other “cytosolic” mitochondria ^26^, whereas ER-associated mitochondria are important for normal insulin signaling ^27^. We hypothesized that such organelle heterogeneity could be explained by the identity of their organelle interacting partner. Therefore, we divided our data according to relative organelle interactions with ER, glycogen, LDs, or mitochondria (Figure 4G–H, Figure S5D); surprisingly, most mitochondria that were either isolated or in contact with LDs had lower ^13^C/^12^C ratios, whereas mitochondria in contact with ER or glycogen continued to display a bi-modal histogram distribution (Figure 4G, Figure S5D). In contrast, most LDs with ER contacts had lower ^13^C/^12^C ratios, whereas LD with glycogen contacts had significantly higher ^13^C/^12^C ratios, again supporting the notion that glycogenesis occurs at the vicinity of LDs (Figure 4H, Figure S5D). Notably, none of these differences were associated with changes in organelle size, and all types of LD and mitochondria achieved similar levels of ^13^C enrichment after 4 hours (Figure 4G–H, Figure S5C, and Figure S6A), thus indicating that organelle 13C flux heterogeneity occurs at the early stages of glycogenesis and that organelle ^13^C enrichment is partially explained by the identity of its interacting organelle partner.

In the liver, mitochondria-ER contact sites are implicated in glucose sensing, insulin signaling, and lipid transfer to sustain normal cell function ^27–29^; moreover, a recent study identified mitochondria wrapped by rough ER sheets that are associated with ApoB/VLDL synthesis and secretion ^10^. Therefore, we used spatial analysis to quantify the total area of each individual mitochondria occupied by ER contacts and found that that mitochondria with high ^13^C enrichment levels had significantly more interaction with ER regions (Figure 4I). This data suggests that *i)* mitochondria rich in ER contacts are associated with higher fractional turnover rates derived specifically from ^13^C-glucose metabolism, and ii) that hepatocyte organelle ^13^C enrichment is time- and organelle-dependent and may be (at least partially) correlated with the identity of organelle-interacting partners.

## Limitations of this study

In this study we introduce a multi-modal analysis pipeline to quantify nutrient metabolism and channeling across the mesoscale, from whole animals to single cells. This is achieved by combining *in vivo* measurements of glucose oxidation and metabolism with MIMS-EM imaging and spatial annotation to map the flux of glucose-derived ^13^C into distinct cellular and sub-cellular compartments. MIMS-EM is a very expensive and time-consuming technique, which limits sample throughput. Moreover, due to the physics of stable isotope imaging and detection of MIMS, MIMS-EM is unable to identify the molecular identity of most molecules associated with the spatial patterns of ^13^C distribution, except perhaps glycogen and saturated fatty acids that form lipid droplets (i.e., triacylglycerol and cholesterol esters). To minimize the impact of these factors on our study, we performed MIMS-EM on tissues from at least n=3 animals infused with different doses of glucose for up to 4 hours and focused on hepatocytes due to their well-established role in glucose storage and homeostasis processes. This allowed us to analyze hundreds of cells and thousands of individual organelles to identify the sub-cellular location of (early) glycogen synthesis and subpopulations of mitochondria that could be involved in hepatocyte lipid signaling ^10^. In the future, we anticipate that incorporation of MALDI-MS and other techniques that are compatible with MIMS-EM imaging (i.e., Click-EM ^30^) will allow for identification of molecular species associated with channeling of nutrients types across spatial and temporal scales.
